# Innate immunity in *Aedes* mosquitoes: from pathogen resistance to shaping the microbiota

**DOI:** 10.1098/rstb.2023.0063

**Published:** 2024-05-06

**Authors:** Bretta Hixson, Robin Chen, Nicolas Buchon

**Affiliations:** Department of Entomology, Cornell University College of Agriculture and Life Sciences, Ithaca, 14853, NY, USA

**Keywords:** innate immunity, antimicrobial peptides, microbiota, entomopathogens, mosquito *Aedes aegypti*, Toll and Imd pathways

## Abstract

Discussions of host–microbe interactions in mosquito vectors are frequently dominated by a focus on the human pathogens they transmit (e.g. *Plasmodium* parasites and arboviruses). Underlying the interactions between a vector and its transmissible pathogens, however, is the physiology of an insect living and interacting with a world of bacteria and fungi including commensals, mutualists and primary and opportunistic pathogens. Here we review what is known about the bacteria and fungi associated with mosquitoes, with an emphasis on the members of the *Aedes* genus. We explore the reciprocal effects of microbe on mosquito, and mosquito on microbe. We analyse the roles of bacterial and fungal symbionts in mosquito development, their effects on vector competence, and their potential uses as biocontrol agents and vectors for paratransgenesis. We explore the compartments of the mosquito gut, uncovering the regionalization of immune effectors and modulators, which create the zones of resistance and immune tolerance with which the mosquito host controls and corrals its microbial symbionts. We examine the anatomical patterning of basally expressed antimicrobial peptides. Finally, we review the relationships between inducible antimicrobial peptides and canonical immune signalling pathways, comparing and contrasting current knowledge on each pathway in mosquitoes to the model insect *Drosophila melanogaster*.

This article is part of the theme issue ‘Sculpting the microbiome: how host factors determine and respond to microbial colonization’.

## 1. Introduction

Mosquitoes are prolific vectors of disease-causing human pathogens. Mosquito-borne arboviruses are transmitted to humans hundreds of millions of times per year, and *Plasmodium* parasites transmitted by mosquitoes cause hundreds of thousands of deaths annually [1]. Consequently, much of the research into host–microbe relationships in mosquitoes is centred on their interactions with human pathogens. It is important, however, not to overlook their interactions with other types of microbes, including environmental bacteria and fungi—especially those that populate the gut. Mosquitoes and their microbiota mutually shape one another: microbes facilitate mosquito development, interface with gut function and fundamentally alter vector competence. Mosquitoes select and corral their microbiota, and maintain a system of both basally expressed and inducible immune defences to check microbial overgrowth and infection. The inducible branch of the immune system is capable of specific recognition of, and tailored responses to, bacterial and fungal challenges.

In this mini-review, we will examine host–microbiota interactions in mosquitoes of the *Aedes* genus, which are prolific vectors of viral pathogens. We will explore the composition of the microbiota, briefly summarize the intrinsic and extrinsic factors that shape it, and describe some of the key ways in which the microbiota interface with and influence the physiology of the mosquito host. We will examine some potential applications of bacteria and fungi for the control of mosquito-borne disease transmission, such as paratransgenesis and the use of entomopathogenic fungi for biological pest management. We will conclude with a discussion of the important reciprocal interactions of *Aedes* mosquitoes with their symbiont microbes—from immune tolerance of commensals and mutualists to the targeted expression of antimicrobial effectors in defence against systemic invasion.

## 2. The mosquito microbiota: what are the microbes associated with *Aedes*?

The mosquito microbiota is diverse; mosquito-microbe associations have been reported with bacteria, fungi, viruses and protists [2–5]. The gut is the main site of colonization, but microbial associations with other tissues, such as salivary glands and reproductive organs, have also been reported (table 1). In this section, we will focus primarily on the bacterial inhabitants of the guts of *Aedes* mosquitoes and discuss factors that influence the composition of the gut community and its roles in mosquito physiology and vector competence.

**Table 1.** Examples of bacterial genera isolated from adult *Aedes* mosquitoes.

bacterial phylum	bacterial genus	mosquito species	mosquito tissue	mosquito location	references
Pseudomonadota	*Acinetobacter*	*Ae. aegypti*	whole mosquito	Madagascar	Zouache *et al*. [6]
			reproductive organs	laboratory colony	Mancini *et al*. [7]
		*Ae. albopictus*	whole mosquito	Madagascar	Moro *et al*. [8]; Zouache *et al*. [6]
			midgut, salivary gland	laboratory colony	Zouache *et al*. [9]
			midgut	India	Yadav *et al*. [10,11]
				Brazil	David *et al*. [12]
	*Aeromonas*	*Ae. aegypti*	midgut	India	Yadav *et al*. [10]
				Brazil	David *et al*. [12]
	*Agrobacterium*	*Ae. albopictus*	whole mosquito	Madagascar	Zouache *et al*. [6]
	*Alcaligenes*	*Ae. albopictus*	midgut	India	Yadav *et al*. [10]
	*Ancylobacter*	*Ae. aegypti*	midgut	Brazil	David *et al*. [12]
	*Asaia*	*Ae. aegypti*	whole mosquito	Madagascar	Zouache *et al*. [6]
			midgut, ovary	laboratory colony	Gusmão *et al*. [13]
			gut	laboratory colony	Mancini *et al*. [7]
		*Ae. albopictus*	whole mosquito	Madagascar	Moro *et al*. [8]; Zouache *et al*. [6]
			gut, salivary gland	laboratory colony	Mancini *et al*. [7]
	*Bradyrhizobium*	*Ae. aegypti*	midgut	Brazil	David *et al*. [12]
		*Ae. albopictus*	whole mosquito	Madagascar	Zouache *et al*. [6]
	*Buttiauxella*	*Ae. aegypti*	midgut	Brazil	David *et al*. [12]
	*Chromobacterium*				
	*Citrobacter*	*Ae. albopictus*	whole mosquito	Madagascar	Moro *et al*. [8]; Zouache *et al*. [6]
	*Comamonas*	*Ae. albopictus*	midgut	laboratory colony	Zouache *et al*. [9]
	*Cupriavidus*	*Ae. aegypti*	gut, salivary gland	laboratory colony	Mancini *et al*. [7]
		*Ae. albopictus*	gut, reproductive organs, salivary gland	laboratory colony	Mancini *et al*. [7]
	*Delftia*	*Ae. aegypti*	midgut	Brazil	David *et al*. [12]
		*Ae. albopictus*	whole mosquito	Madagascar	Zouache *et al*. [6]
			midgut	India	Yadav *et al*. [11]
	*Enterobacter*	*Ae. aegypti*	whole mosquito	Madagascar	Zouache *et al*. [6]
			midgut	India	Yadav *et al*. [10]
		*Ae. albopictus*	whole mosquito	Madagascar	Moro *et al*. [8]; Zouache *et al*. [6]
			midgut	India	Yadav *et al*. [10,11]
	*Escherichia*	*Ae. aegypti*	gut	laboratory colony	Mancini *et al*. [7]
		*Ae. albopictus*	gut, salivary gland	laboratory colony	Mancini *et al*. [7]
	*Haematobacter*	*Ae. albopictus*	whole mosquito	Madagascar	Moro *et al*. [8]
	*Halomonas*	*Ae. aegypti*	midgut	Brazil	David *et al*. [12]
	*Herbaspirillum*	*Ae. aegypti*	midgut	Brazil	David *et al*. [12]
		*Ae. albopictus*	whole mosquito	Madagascar	Zouache *et al*. [6]
	*Janthinobacterium*	*Ae. aegypti*	midgut	Brazil	David *et al*. [12]
	*Klebsiella*	*Ae. aegypti*	abdomen	laboratory colony	Terenius *et al*. [14]
			midgut	India	Yadav *et al*. [10]
		*Ae. albopictus*	whole mosquito	Madagascar	Moro *et al*. [8]
			midgut	India	Yadav *et al*. [10,11]
	*Kluyvera*	*Ae. aegypti*	midgut	laboratory colony	Gusmão *et al*. [13]
	*Methylobacterium*	*Ae. aegypti*	midgut	Brazil	David *et al*. [12]
	*Morganella*	*Ae. aegypti*	midgut	Brazil	David *et al*. [12]
	*Neisseria*	*Ae. albopictus*	whole mosquito	Madagascar	Moro *et al*. [8]
	*Nevskia*	*Ae. aegypti*	midgut	Brazil	David *et al*. [12]
	*Ochrobactrum*	*Ae. aegypti*	reproductive organs, salivary gland	laboratory colony	Mancini *et al*. [7]
	*Pantoea*	*Ae. aegypti*	abdomen	laboratory colony	Terenius *et al*. [14]
			midgut, ovary	laboratory colony	Gusmão *et al*. [13]
			midgut	India	Yadav *et al*. [10]
		*Ae. albopictus*	whole mosquito	Madagascar	Moro *et al*. [8]; Zouache *et al*. [6]
	*Pseudomonas*	*Ae. aegypti*	whole mosquito	Madagascar	Zouache *et al*. [6]
			gut	Kenya	Osei-Poku *et al*. [15]
			midgut	India	Yadav *et al*. [10]
				Brazil	David *et al*. [12]
			gut, reproductive organs, salivary gland	laboratory colony	Mancini *et al*. [7]
		*Ae. albopictus*	whole mosquito	Madagascar	Moro *et al*. [8]; Zouache *et al*. [6]
			midgut	India	Yadav *et al*. [10,11]
			gut, salivary gland	laboratory colony	Mancini *et al*. [7]
	*Rhizobium*	*Ae. albopictus*	whole mosquito	Madagascar	Zouache *et al*. [6]
	*Rickettsia*	*Ae. albopictus*	whole mosquito	Madagascar	Zouache *et al*. [6]
	*Serratia*	*Ae. aegypti*	abdomen	laboratory colony	Terenius *et al*. [14]
			ventral diverticulum, midgut	laboratory colony	Gusmão *et al*. [13,16]
			gut, salivary gland	laboratory colony	Mancini *et al*. [7]
		*Ae. albopictus*	gut, reproductive organs, salivary gland	laboratory colony	Mancini *et al*. [7]
	*Shigella*	*Ae. aegypti*	gut	laboratory colony	Mancini *et al*. [7]
		*Ae. albopictus*	whole mosquito	Madagascar	Zouache *et al*. [6]
			gut, salivary gland	laboratory colony	Mancini *et al*. [7]
	*Skermanella*	*Ae. albopictus*	whole mosquito	Madagascar	Moro *et al*. [8]
	*Sphingomonas*	*Ae. aegypti*	abdomen	laboratory colony	Terenius *et al*. [14]
			midgut	Brazil	David *et al*. [12]
			gut, reproductive organs, salivary gland	laboratory colony	Mancini *et al*. [7]
		*Ae. albopictus*	whole mosquito	Madagascar	Moro *et al*. [8]
			gut, reproductive organs, salivary gland	laboratory colony	Mancini *et al*. [7]
	*Stenotrophomonas*	*Ae. aegypti*	midgut	India	Yadav *et al*. [10]
				Brazil	David *et al*. [12]
			reproductive organs, salivary gland	laboratory colony	Mancini *et al*. [7]
		*Ae. albopictus*	whole mosquito	Madagascar	Zouache *et al*. [6]
			midgut	India	Yadav *et al*. [11]
	*Undibacterium*	*Ae. aegypti*	midgut	Brazil	David *et al*. [12]
	*Vibrio*	*Ae. aegypti*	midgut	Brazil	David *et al*. [12]
	*Wolbachia*	*Ae. albopictus*	midgut, salivary gland, ovary	laboratory colony	Zouache *et al*. [9]
			reproductive organs	laboratory colony	Mancini *et al*. [7]
	*Xanthomonas*	*Ae. albopictus*	whole mosquito	Madagascar	Moro *et al*. [8]
	*Yersinia*	*Ae. aegypti*	midgut	Brazil	David *et al*. [12]
	*Yokenella*	*Ae. albopictus*	whole mosquito	Madagascar	Zouache *et al*. [6]
	*Zymobacter*	*Ae. aegypti*	gut	Kenya	Osei-Poku *et al*. [15]
					
Actinomycetota	*Arsenicicoccus*	*Ae. albopictus*	whole mosquito	Madagascar	Moro *et al*. [8]
	*Arthrobacter*	*Ae. albopictus*	whole mosquito	Madagascar	Moro *et al*. [8]
	*Cellulosimicrobium*	*Ae. albopictus*	whole mosquito	Madagascar	Moro *et al*. [8]
	*Curtobacterium*	*Ae. albopictus*	whole mosquito	Madagascar	Moro *et al*. [8]
	*Kocuria*	*Ae. albopictus*	whole mosquito	Madagascar	Moro *et al*. [8]
			midgut	India	Yadav *et al*. [11]
	*Leucobacter*	*Ae. albopictus*	whole mosquito	Madagascar	Moro *et al*. [8]
	*Microbacterium*	*Ae. albopictus*	whole mosquito	Madagascar	Moro *et al*. [8]
	*Micrococcus*	*Ae. aegypti*	midgut	India	Yadav *et al*. [10]
		*Ae. albopictus*	midgut	India	Yadav *et al*. [11]
	*Propionibacterium*	*Ae. aegypti*	midgut	Brazil	David *et al*. [12]
	*Streptomyces*	*Ae. albopictus*	whole mosquito	Madagascar	Moro *et al*. [8]
					
Firmicutes	*Aerococcus*	*Ae. albopictus*	midgut	India	Yadav *et al*. [11]
	*Bacillus*	*Ae. aegypti*	whole mosquito	Madagascar	Moro *et al*. [8]; Zouache *et al*. [6]
			abdomen	laboratory colony	Terenius *et al*. [14]
			ventral diverticulum, midgut	laboratory colony	Gusmão *et al*. [13,16]
			midgut	India	Yadav *et al*. [10]
		*Ae. albopictus*	whole mosquito	Madagascar	Zouache *et al*. [6]
			midgut	India	Yadav *et al*. [10,11]
	*Clostridium*	*Ae. aegypti*	midgut	Brazil	David *et al*. [12]
	*Enterococcus*	*Ae. aegypti*	abdomen	laboratory colony	Terenius *et al*. [14]
			midgut	laboratory colony	Gusmão *et al*. [13]
	*Lysinibacillus*	*Ae. aegypti*	midgut	India	Yadav *et al*. [10]
		*Ae. albopictus*	midgut	India	Yadav *et al*. [10]
	*Paenibacillus*				
	*Planococcus*	*Ae. albopictus*	whole mosquito	Madagascar	Moro *et al*. [8]
	*Staphylococcus*	*Ae. aegypti*	whole mosquito	Madagascar	Zouache *et al*. [6]
			midgut	India	Yadav *et al*. [10]
		*Ae. albopictus*	whole mosquito	Madagascar	Moro *et al*. [8]; Zouache *et al*. [6]
			midgut	India	Yadav *et al*. [10,11]
Bacteroidota	*Chryseobacterium*	*Ae. aegypti*	gut	Kenya	Osei-Poku *et al*. [15]
		*Ae. albopictus*	midgut	India	Yadav *et al*. [11]
	*Elizabethkingia*	*Ae. aegypti*	abdomen	laboratory colony	Terenius *et al*. [14]
			reproductive organs	laboratory colony	Mancini *et al*. [7]
		*Ae. albopictus*	midgut	India	Yadav *et al*. [10]
	*Myroides*	*Ae. aegypti*	midgut	Brazil	David *et al*. [12]
Deinococcota	*Deinococcus*	*Ae. albopictus*	whole mosquito	Madagascar	Moro *et al*. [8]

### (a) The intrinsic and extrinsic factors that shape the composition of *Aedes* microbiota

Bacterial genera associated with *Aedes* mosquitoes are primarily from the Gram-negative phylum Pseudomonadota and the Gram-positive phyla Actinomycetota and Firmicutes (table 1), but the exact composition of the microbiome is highly variable. Fungi associated with *Aedes* mosquitoes are predominantly members of the phyla Ascomycota and Basidiomycota, including both yeasts and filamentous fungi (reviewed by Malassigné *et al*. [4]). Protists (e.g. *Ascogregarina*) and viruses found in mosquitoes are beyond the scope of this review and are described or reviewed elsewhere [2,3]. The identities of *Aedes* midgut microbes have been studied in the context of a wide range of factors. Variation in the microbiota has been associated with intrinsic characteristics such as age. In adult *Aedes aegypti* and *Aedes albopictus*, older mosquitoes harbour more *Asaia* and *Wolbachia*, respectively, compared to younger mosquitoes [12,17]. Changes in immune regulation during mosquito ageing have been suggested to underlie age-associated changes in the composition of the microbiota [5], but this has not, to our knowledge, been experimentally demonstrated. Sex, another intrinsic characteristic, also appears to play a role in the composition of the gut community. In one study, the midgut microbiota of adult male *Ae. albopictus* was dominated by *Enterobacter*, *Pantoea* and *Pseudomonas*, while the guts of their sugar-fed female counterparts were dominated by *Acinetobacter*, *Enterobacter* and *Micrococcus* [11]. The microbiota composition also differs between mosquito species. For example, *Ae. albopictus* and *Anopheles gambiae* reared together as larvae harbour some different species of microbes as adults despite identical environment and diet [18].

While intrinsic characteristics are at least partially determinative of the gut community, variations between mosquito populations are thought to be more dependent on extrinsic factors associated with the environment. Mosquitoes of the same species collected from different geographical locations are often dominated by different species of bacteria. For example, in one study, the microbiota of *Ae. albopictus* collected from Pradesh, India was dominated by *Enterobacter* and *Bacillus*, whereas *Acinetobacter* and *Pseudomonas* were dominant in *Ae. albopictus* collected from Tezpur, India [10,11]. In studies of laboratory populations, mosquitoes of the same species and strain (*Ae. aegypti* Rockefeller strain) maintained in different laboratories harboured different microbiota compositions whereas different species of *Aedes* mosquitoes (*Ae. aegypti* versus *Ae. albopictus*) or different colonies of the same species of mosquitoes (*Ae. aegypti* collected from different countries and continents) maintained in the same laboratory were found to have similar microbial compositions [13,14,19,20]. Field-collected mosquitoes harbour greater microbial diversity than their laboratory-reared counterparts, probably as a reflection of the greater diversity of microbes present in the field [21]. In summary, both intrinsic factors (e.g. age and sex) and extrinsic factors (e.g. environment) influence microbiota composition in *Aedes* mosquitoes.

### (b) Mechanisms of microbiota acquisition

Mosquito gut microbes are mainly acquired from the environment. Larvae hatch without extracellular microbes in the gut [22], and the diversity of microbes associated with the mosquito microbiota represents a subset of the microbial diversity in the larval environment [5]. However, an indirect form of vertical transmission of gut microbes has been documented. In laboratory conditions, oviposition by *Ae. aegypti* reduced microbial diversity in the larval water by mechanically introducing bacteria such as *Elizabethkingia*, which accelerated larval development and increased larval fitness [23]. Some parts of the larval microbiota persist trans-stadially to adulthood but most are lost during metamorphosis and are reacquired by adults imbibing water from the larval environment [5,22,24]. New species may, in theory, be acquired by adults through nectar feeding [25], although we are unaware of any documentation of this phenomenon. Blood feeding increases bacterial load in the mosquito midgut owing to the nutrient-rich environment in the midgut supporting bacterial proliferation and precipitates changes in the bacterial composition of the gut community owing to differences in the abilities of different bacteria to tolerate the oxidative stress associated with blood digestion [5,14]. The blood bolus and peritrophic matrix are excreted at the conclusion of blood meal digestion, but blood-feeding exerts some effects on the microbiota which persist after the blood bolus is excreted. The source of the blood meal can also change microbiota composition in mosquitoes. The midgut microbiota of *Ae. aegypti* which fed on human, rabbit and chicken blood were, respectively, dominated by *Serratia*, *Elizabethkingia* and *Chryseobacterium* 3 days after blood feeding*.* At 7 days after feeding on human blood, the microbiota composition remained different compared to sugar-fed mosquitoes [26]. In summary, the larval environment is the main source of mosquito larval microbiota, some of which are retained or reacquired by adults. Subsequent changes in the microbiota may occur through blood and nectar feeding.

### (c) Gut microbiota and the mosquito lifecycle

**Figure 1.**
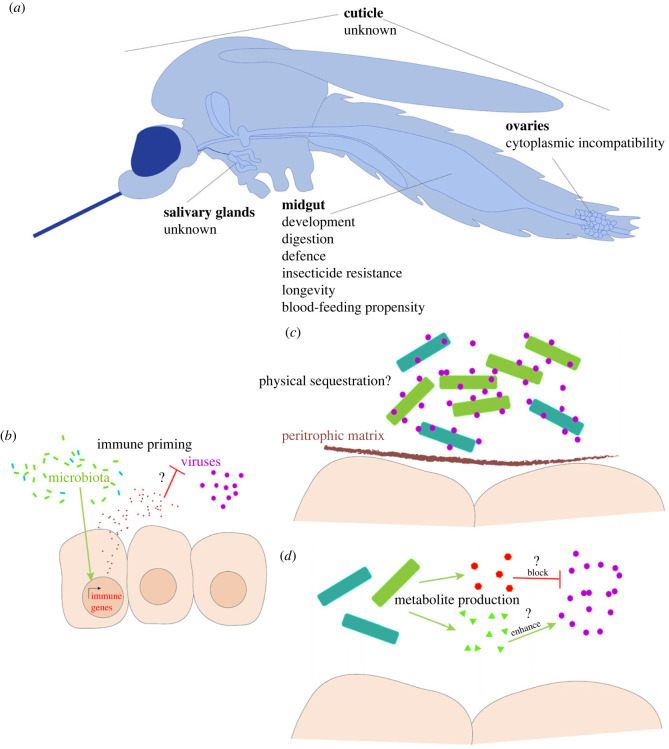
Roles of the microbiota in mosquito life history and vector competence. (*a*) Known functions of the microbiota in different tissues and locations in mosquitoes. (*b*) Microbiota-induced immune gene expression reduces viral load via unknown mechanisms. (*c*) Arbovirus sequestration by the microbiota reduce the number of infective viral particles. (*d*) Metabolites produced by the microbiota enhance or inhibit arbovirus infection. Question marks indicate where the mechanisms remain unknown.

The gut microbiota plays important, often essential, roles in the lifecycle and physiology of the mosquito. Mosquitoes that were axenic throughout development show reduced adult size and lifespan compared to colonized mosquitoes, while mosquitoes that were transiently colonized during larval development did not [27,28]. Mosquito development requires the supplementation of riboflavin, which mosquitoes themselves cannot synthesize, by the gut microbiota [29]. Axenic mosquitoes exposed to light are unable to develop past the first instar owing to light-dependent degradation of dietary riboflavin. Development can be rescued by gnotobiotic colonization with individual bacterial or fungal species [22]. Transcriptomic analysis of germ-free and gnotobiotic mosquito larvae suggests that folate supplementation, lipid digestion and protein digestion by gut microbiota may also be important for mosquito larval development [28]. Another role of the mosquito gut microbiota is the contribution to blood meal digestion. Antibiotic treatments prior to blood feeding reduce haemolysis, protein digestion and fecundity in *Ae. aegypti* [30]. Axenic *Ae. aegypti* obtained without the use of antibiotics, however, show reduced lifespan but similar fecundity compared to mosquitoes with intact microbiota [27]. The discrepancy in fecundity may be owing to antibiotic toxicity and/or dysbiosis from the incomplete clearance of bacteria. The mosquito gut microbiota also inhibits pathogen colonization. For example, in gnotobiotic *Ae. aegypti* that are colonized by *Cedecea*, subsequent colonization by *Serratia* is reduced compared to axenic mosquitoes [31]. This is attributed to competitive exclusion by the authors, but we cannot rule out other mechanisms such as the activation of mosquito immune defences by *Cedacea*—a phenomenon known as immune-priming. Members of the microbiota can also alter blood-feeding behaviour in mosquitoes. *Serratia*, for example, reduces the propensity of *Ae. aegypti* to blood-feeding compared to uninfected and antibiotic-treated mosquitoes [31]. In the context of blood-feeding, resident gut microbiota is also believed to protect the mosquito against pathogenic damage to the gut epithelium by stimulating the secretion of a peritrophic matrix (PM). The PM is a physical barrier consisting of chitin and protein that separates the contents of the midgut lumen from the epithelium. Antibiotic-treated mosquitoes show reduced antimicrobial peptide (AMP) expression and disrupted PM formation, potentially increasing the vulnerability of the mosquitoes to pathogenic infection [32,33]. The interplays between the microbiota and mosquito immune pathways are discussed in more detail later in this review. Gut microbiota was also implicated in mediating resistance against the insecticide permethrin in *Ae. aegypti* [34]. The mechanism behind this microbiota-mediated insecticide resistance remains unknown, but could potentially involve detoxification of the insecticide by the microbiota. Overall, known roles of the mosquito gut microbiota include development, digestion, longevity, protection from pathogens, changes in blood-feeding propensity and insecticide resistance (figure 1*a*).

### (d) Microbial effects on vector competence

Members of the microbiota can alter the vector competence of mosquitoes to transmit arboviruses and parasites. This effect is species-specific and can be positive or negative. Midgut colonization by *Chromobacterium*, *Paenibacillus* and *Proteus*, for example, has been shown to reduce Dengue virus (DENV) load in *Ae. aegypti* [35,36]. This phenomenon may be associated with the induction of immune gene expression by the presence of the bacteria, although how this results in reduced viral load remains unknown [36]. Exposure of DENV to *Chromobacterium* biofilm *in vitro* reduced DENV infectivity in a vertebrate cell line. This effect was abolished when heat-treated biofilm was used and the effect was absent for biofilms from other bacteria or in planktonic *Chromobacterium*, suggesting the presence of *Chromobacterium* biofilm-specific heat-sensitive metabolites that reduce DENV infectivity through unknown mechanisms [35]. In another study, La Crosse virus titer was reduced by incubating the virus with isolates of *Ae. albopictus* gut bacteria and removing the bacteria prior to performing plaque assay, possibly indicating adhesion of viral particles to bacteria. Together, bacterial proliferation and PM formation post-bloodmeal could allow some virions to adhere to the bacteria and be excluded by the PM in the endoperitrophic space, restricting access to the gut epithelium [37]. The endosymbiont *Wolbachia* reduces vector competence for DENV, yellow fever virus, West Nile virus and Chikungunya virus but the mechanisms remain unknown; immune-priming and/or resource competition have been proposed to play a role [38,39].

While some microbes alter the vector competence of mosquitoes negatively, others have been shown to increase it. For example, the presence of *Serratia odorifera* with DENV in the bloodmeal, was shown to increase DENV prevalence, but not viral load, in *Ae. aegypti*. A 40 kDa *Serratia* protein (P40) was found to interact with the midgut brush border of *Ae. aegypti* through binding to prohibitin and porin, possibly affecting viral entry. Three other *Serratia* proteins (19, 29, 36 kDa) interact with DENV directly. Whether these interactions alter DENV infectivity remains unknown and requires future research to determine [40]. *Serratia marcescens* was shown to increase DENV, Zika virus and Sindbis virus prevalence and viral load in another study via degradation of brush border mucins [41]. *Talaromyces*, a fungus associated with *Ae. aegypti* collected from DENV-endemic Puerto Rico, was found to increase DENV prevalence and load in the mosquito by reducing midgut trypsin expression [42]. The mechanism of trypsin downregulation by *Talaromyces* and how this results in increased vector competence remain unknown.

The effects of the mosquito microbiota on vector competence for arboviruses are summarized in figure 1*b–d*. Overall, the literature indicates that modulation of vector competence of *Aedes* mosquitoes by the microbiota is variable and depends on the species of mosquito, gut microbes and arbovirus in question. The mechanisms of these tri-partite interactions are complex and remain poorly understood, requiring further studies to elucidate. Understanding these mechanisms could potentially lead to the development of new tools that can reduce mosquito vector competence against arboviruses to limit disease transmission.

## 3. Using microbes to develop new transmission control strategies in *Aedes*

As seen in the previous section, the bacteria and fungi that interact with mosquitoes in general, and with *Aedes* specifically, have the ability to influence mosquito lifespan, fitness and disease transmission. Therefore, researchers have used these abilities to implement microbial-based strategies to reduce the transmission of mosquito-vectored diseases. These strategies include: (i) mosquito population reduction by the use of bio insecticides, such as *Bacillus thuringiensis* and entomopathogenic fungi; and (ii) population replacement by mosquitoes with reduced vector competence through association with endosymbiotic bacteria such as *Wolbachia* or genetically manipulated gut microbes (paratransgenesis) [43].

### (a) Entomopathogenic microbes as bio insecticides

#### (i) *Bacillus* *thuringiensis*

*Bacillus thuringiensis israelensis* (Bti) is a Gram-positive entomopathogenic soil bacterium that is applied to water to kill mosquito larvae. Sporulating Bti produces δ-endotoxins (Cry toxins) that form pores on the enterocyte cell membranes when ingested by the larvae, resulting in cell death and loss of gut barrier function, paralysing and killing the larvae [44]. Bti killed *Ae. aegypti* and *Ae. albopictus* larvae in the field but long-term use could result in the development of resistance against the Cry toxins in these mosquitoes [43].

#### (ii) Entomopathogenic fungi

Entomopathogenic fungi (e.g. *Beauvaria bassiana* and *Metarhizium robertsii*) are mainly used to kill adult mosquitoes [43]. Fungal conidia are sprayed onto foliage. When encountered by mosquitoes, the conidia attach to the cuticle where they germinate; the germ tube penetrates the cuticle by enzymatic digestion and physical pressure, gaining access to the haemocoel where the fungus replicates and kills the mosquito by a combination of toxin production and nutrient depletion [45]. The cuticular route of infection allows entomopathogenic fungi to infect insects such as adult mosquitoes without the need for ingestion. The effectiveness and challenges of using entomopathogenic fungi against mosquitoes is reviewed elsewhere [46].

### (b) Using mosquito-associated microbes to reduce pathogen transmission

#### (i) Wolbachia

*Wolbachia* is a Gram-negative intracellular symbiotic bacterium found in many insects. It can induce cytoplasmic incompatibility (CI) when infected males mate with uninfected females, resulting in inviable eggs and population reduction. However, infected females can mate with both infected and uninfected males to produce viable eggs infected with *Wolbachia*, thereby increasing *Wolbachia* prevalence in the mosquito population over time and decreasing arbovirus transmission [38,43]. *Wolbachia* CI factors CifA and CifB in the male *Drosophila* germline induce CI, while CifA expression from the female germline rescues CI [47]. The mechanisms behind CI induction by Cifs are currently unknown. A *Wolbachia*-based strategy to control arbovirus transmission has achieved success and is reviewed elsewhere [48].

#### (ii) Paratransgenesis

An ideal target for paratransgenesis is a symbiont that readily colonizes and stably associates with the mosquito while causing no pathology or fitness costs and possessing the ability to be transmitted vertically and horizontally. *Asaia bogorensis*, a Gram-negative bacterium in the family Acetobacteraceae, is an example of a good candidate for paratransgenesis owing to its: (i) broad host range (identified in wild populations of *Aedes*, *Anopheles* and *Culex* species); (ii) stable colonization of mosquito midguts (the site of blood meal digestion and pathogen entry), salivary glands (the site of pathogen exit), and reproductive organs (vertical and venereal transmission to spread through the mosquito population); and (iii) cultivability *in vitro* (ease of genetic manipulation) [49]. Shane *et al*. transformed *Asia* with plasmids encoding scorpine, an antiplasmodial peptide, under the control of blood meal-induced promoters to achieve conditional expression of scorpine in the presence of a blood meal, resulting in the reduction of *Plasmodium* oocyst prevalence and load in *Anopheles stephensi* [50]. Since *Asaia* also colonizes the midgut and salivary glands of *Ae. aegypti,* which are critical locations for arboviral transmission, *Asaia* could potentially be used similarly to express genes that reduce vector competence for arboviruses upon blood feeding [7,51]. *Wolbachia*, with CI, lifespan reduction, and vector competence reduction already in place, would be a great candidate for paratransgenesis to improve its effectiveness. However, *Wolbachia* is an obligate intracellular symbiont that cannot be cultured *in vitro* under cell-free conditions, making *Wolbachia* transformation unfeasible with current methods [52]. Other members of the mosquito microbiota that reduce vector competence for arboviruses (e.g. *Chromobacterium* discussed previously) are also potential candidates for paratransgenesis to increase their efficacy. Bacteria such as *Pantoea* and *Serratia* have also been considered for use in paratransgenesis owing to their prevalence in the mosquito microbiota [49]. Wang *et al*. transformed *Pantoea* to express and secrete antiplasmodial peptides, resulting in reduced oocyst counts in the midgut of *An. gambiae* [53]. *Serratia* is found in the midgut and salivary glands of *Ae. aegypti* and reduces blood-feeding propensity [7,31]. However, since it is also known to increase vector competence for the transmission of multiple arboviruses, careful selection of a strain that does not enhance vector competence is required prior to implementation of a paratransgenic approach [41]. To our knowledge, paratransgenesis of bacterial symbionts has not been implemented in *Aedes* mosquitoes. Future research identifying and testing new and existing paratransgenesis candidates and anti-arboviral effectors for *Aedes* mosquitoes could lead to more effective strategies to control arbovirus transmission.

## 4. How mosquitoes interact with microbes: the example of *Aedes* antimicrobial peptides

Host–microbe interactions between mosquitoes and their bacterial and fungal symbionts are bi-directional: microbes alter mosquito physiology and vector competence; reciprocally, mosquitoes employ resistance and immune tolerance to shape their associated microbial communities. Mosquitoes resist bacterial and fungal infection through a combination of cellular and humoral mechanisms. Prominent among these are (i) phagocytosis and nodulation by haemocytes, (ii) complement-mediated lysis and melanization, (iii) Duox-mediated reactive oxygen species production in the midgut, and (iv) the expression of AMPs. The first three of these four mechanisms are well reviewed elsewhere [54,55], but recent publications have shed new light on the fourth. We will therefore focus our discussion on antimicrobial peptides, with specific attention to *where* in the body they are expressed, under what circumstances and in what manner they are induced, and *how* their induction is controlled.

### (a) Antimicrobial peptides

AMPs are small, secreted, typically cationic effectors, most of which interfere with the plasma membrane integrity of pathogens [56]. Mosquitoes express AMPs belonging to a number of families including attacins, cecropins, defensins, gambicins and (in *Ae. aegypti*) holotricins. Attacins, cecropins and defensins are widespread among insects, and their modes of action and specificities are reviewed in Hui-Yu *et al.* [57]. In brief, attacins are primarily active against Gram-negative bacteria, defensins are primarily active against Gram-positive bacteria (but with some anti-Gram-negative and anti-fungal activity), and cecropins are generally considered to be active against all three types of pathogen. Gambicin was first discovered in *An. gambiae*, where it was shown to kill bacteria of both Gram types (*Escherichia coli* and *Micrococcus luteus*) and to disrupt the germination and hyphal elongation of a filamentous fungus (*Neurospora crassa*) [58]. The *Ae. aegypti* holotricin (*GRRP*) was identified on the basis of its resemblance to peptides from the coleopteran *Holotrichia diomphalia* with activity against Gram-negative bacteria and fungi [59–61]. To our knowledge, the specific activity of *GRRP* has not been demonstrated.

In addition to the known families of AMPs detailed above, *Ae. aegypti* and *An. gambiae* mosquitoes possess conserved genes encoding secreted peptides which, on the basis of expression patterns and physical attributes (e.g. glycine-richness [62], size, isoelectric point [63]) we strongly suspect of possessing antimicrobial properties. In a previous publication, we designated these glycine-rich candidate AMPs as ‘type D’ (comprising one gene in *An. gambiae* and six paralogues in *Ae. aegypti*) and ‘type F’ (comprising one uncharacterized gene in *Ae. aegypti*—an apparent paralogue of the holotricin *GRRP*—and a pair of paralagous genes in *An. gambiae*) [64]. Pending experimental confirmation of antimicrobial activity, we will hereafter refer to the members of these two groups as ‘putative AMPs’. See table 2 for gene identities (IDs).

**Table 2.** IDs of new putative glycine-rich AMPs.

gene ID	type	species
AAEL001392	F	*Aedes aegypti*
AGAP005889	F	*Anopheles gambiae*
AGAP005888	F	*Anopheles gambiae*
AAEL017380	D	*Aedes aegypti*
AAEL021929	D	*Aedes aegypti*
AAEL025531	D	*Aedes aegypti*
AAEL025126	D	*Aedes aegypti*
AAEL026300	D	*Aedes aegypti*
AAEL017144	D	*Aedes aegypti*
AGAP001508	D	*Anopheles gambiae*

### (b) The mosquito gut corrals symbionts with zones of selection and tolerance

**Figure 2.**
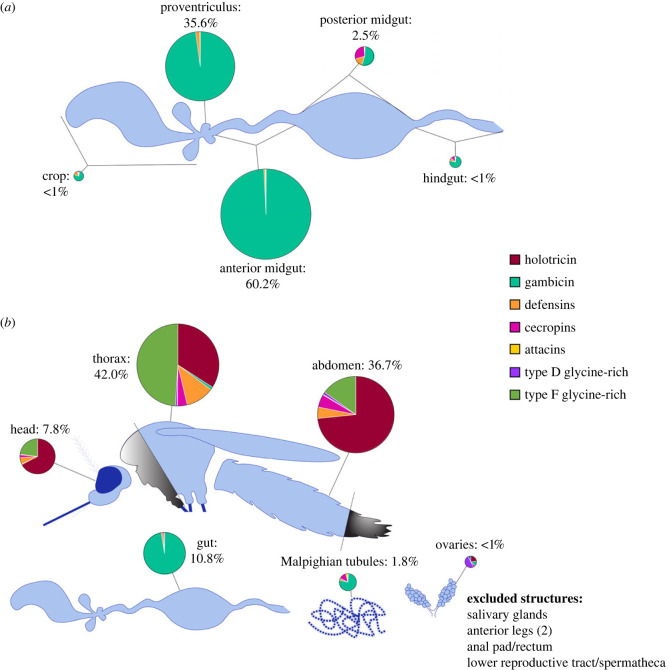
Baseline expression of antimicrobial peptides is unevenly distributed throughout the mosquito gut/body. (*a*) A schematic of antimicrobial peptide (AMP) and putative AMP transcript production in the gut regions of *Aedes aegypti*. (*b*) A schematic of AMP and putative AMP transcript production in the major body parts of *Aedes aegypti*. Percentages are derived from transcripts per million (TPM) values scaled by regional transcriptional yields to estimate the proportion of all AMP/putative AMP transcripts in the whole-body derived from each region/body part. Pie charts are scaled in the same proportion.

The insect gut maintains a unique relationship with environmental microbes. On one hand, it must defend against oral pathogens which may damage the gut epithelium [65,66] and/or infiltrate the haemocoel [67,68]. On the other, it plays host to a community of commensals and mutualists [69]. One possible strategy for balancing these apparently contradictory imperatives is suggested by the transcriptional patterning of AMPs within the *Ae. aegypti* gut, where over 95% of all AMP/putative AMP transcripts are derived from the proventriculus and anterior midgut. The posterior midgut, by contrast, contributes fewer than 3% of the total AMP/putative AMP transcripts in the gut, despite being the source of more than 85% of all gut transcripts genome-wide [70] (figure 2*a*). In the same study, we also found disproportionately high expression of AMP transcripts in the proventriculus and anterior midgut of *An. gambiae* (*s.l.*) as compared to the posterior midgut. Microarray data from *An. gambiae* likewise suggest the concentration of immune function in the anterior regions of the midgut [71]. These observations led us to propose that the proventriculus and anterior midgut of mosquitoes exert selection over the microbial entrants to the gut, and that selected microbes enjoy greater immune tolerance in the posterior midgut, mediated by the immune-modulating effects of amidase peptidoglycan recognition proteins (PGRPs) and the transcription factor *caudal* [70,72]. We derive support for this hypothesis from a subsequent study which found that the crop and midgut of *Ae. aegypti* support significantly different microbial communities, with greater diversity evident in the crop [73]. It should, however, be noted that a similar comparison in the *Ae. albopictus* gut found no substantial difference between the communities inhabiting the two compartments [74].

### (c) Basal antimicrobial peptide expression in the mosquito body

While bacteria and fungi are at least partially tolerated within the lumen of the mosquito gut, the other compartments and tissues of the mosquito body must maintain defences to quickly resist the invasion of the haemocoel, either from opportunistic gut residents, or from microbes that may penetrate the cuticle. AMPs are most frequently studied in the context of infection, but RNAseq studies have demonstrated baseline AMP/putative AMP expression (including the sum of all attacins, cecropins, defensins, gambicin, holotricin and glycine-rich proteins types D and F) ranging from 1639 transcripts per million (TPM) (or 0.16% of all transcripts) to 2606 TPM (0.26%) in female *Ae. aegypti* [64,70] and 5838–5929 TPM (0.58–0.59%) in female *An. gambiae* (*s.l.*) [70]. Transcriptional data from *Ae. aegypti* demonstrate that the anatomical regions of the female body do not contribute equally to the production of these transcripts. When regional investments in the transcription of AMPs/putative AMPs (head: 4197 TPM, thorax: 5467 TPM, abdomen: 3533 TPM, gut: 1953 TPM, Malpighian tubules: 2732 TPM, ovaries: 73 TPM) are scaled by the estimated contributions of each region to the whole-body transcriptome (head: 4.7%, thorax: 19.4%, abdomen: 26.2%, gut: 14.0%, Malpighian tubules: 1.7%, ovaries: 28.8%) we calculate that the head is the source of approximately 8% of all AMP/putative AMP transcripts, the thorax 42%, the abdomen 36%, the gut 11%, the Malpighian tubules 2% and the ovaries 1% (figure 2*b*). It should be noted that the anatomical dataset from which these values were drawn [70] lacks profiles for salivary glands (which were removed from thoraces prior to RNA extraction) and the lower reproductive tract, and therefore their contribution is neglected in this analysis. Transcriptomic profiles from other studies document the expression of some AMPs in *Ae. aegypti* salivary glands, but TPM values vary widely between datasets [75,76]. While tissue-specific transcriptomes are lacking for the segments of the mosquito carcass in this dataset, we presume the AMP transcripts derived from head, thorax and abdomen are most likely produced by the fat body and by haemocytes, and that the resulting peptides are secreted into the haemocoel. AMPs in the gut and Malpighian tubules are presumed to be the product of epithelial cells. It is not clear, at present, whether AMPs produced by these tissues are primarily secreted apically (into the lumen of the gut and/or Malpighian tubules) or basally (into the haemocoel). Future work may shed light into the extent to which AMPs produced by epithelial tissues participate in systemic versus local immune function.

It is notable that the proportions of different AMP/putative AMP transcripts expressed in each body part are not uniform. Transcripts for the two highest expressed peptides in *Ae. aegypti*, *GRRP* and AAEL017144 (a type D glycine-rich peptide), are derived almost exclusively from the head, thorax, and abdomen of the mosquito, while the third (*GAM1*) is expressed almost exclusively in the gut and Malpighian tubules. The Malpighian tubules are the sole source of transcripts for the attacin peptide, *ATT* (figure 2). The cecropin *CECD* is likewise expressed almost exclusively in the Malpighian tubules at baseline [70]. Lacking comparable transcriptomic data for other mosquito species, we are unable to generalize the expression patterns we have described beyond *Ae. aegypti*. Likewise, in the absence of anatomical data from challenged mosquitoes, we are unable to assess how the relative contribution of each body part to overall systemic AMP expression may change in the context of infection. Further work is required to fill these knowledge gaps.

### (d) Antimicrobial peptide induction

**Figure 3.**
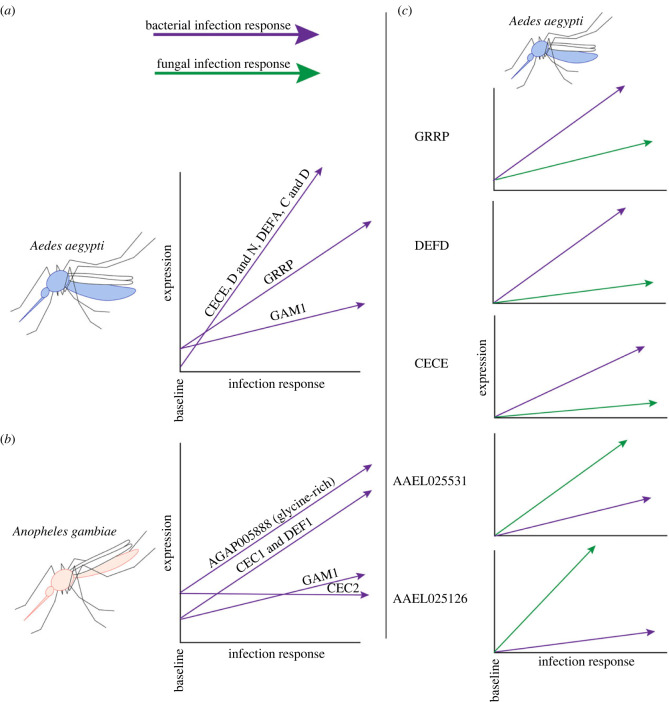
Expression trajectories of highly expressed/induced antimicrobial peptides (AMPs) in mosquitoes systemically infected with bacteria and fungi. (*a,b*) Canonical AMPs are distinguished by variable baseline expression levels and variable amplitudes of upregulation. (*c*) In *Aedes aegypti*, immune inducible AMPs/putative AMPs display different expression trajectories following systemic challenge with bacterial versus fungal challenge. Trajectories in (*a,b*) are generalized from those observed in an RNAseq experiment across multiple infections with multiple Gram-negative and Gram-positive pathogens. Trajectories in (*c*) are modelled after an RNAseq experiment comparing the transcriptomes of mosquitoes infected with *Erwinia carotovora carotovora 15* (a virulent Gram-negative bacterium) and *Candida albicans* (a moderately virulent yeast) which evoke comparable genome-wide transcriptional effects in *Ae. aegypti*.

In contrast to other mechanisms of antimicrobial defence (e.g. the melanization cascade, which is activated post-translationally) AMP-mediated defence is entirely regulated at the level of expression. AMPs and putative AMPs may be loosely grouped by characteristic expression patterns. Some (e.g. the *An. gambiae* gene *CEC2*) are robustly expressed at baseline and not induced by infection. Some are robustly expressed at baseline, but still respond to infection either moderately (e.g. gambicin in both *Ae. aegypti* and *An. gambiae*, and the holotricin *GRRP* in *Ae. aegypti*) or dramatically (e.g. *CEC1* and *DEF1* in *An. gambiae*). Still others are minimally expressed at baseline, but may be upregulated by between two and three orders of magnitude upon infection. Multiple cecropins, defensins and glycine-rich putative AMPs in *Ae. aegypti* fit the latter pattern ([64]; figure 3*a,b*).

Among the infection-responsive AMPs and putative AMPs, there is a diversity of responses to different types of immune challenge. The most notable of these differences are observed in the response to bacterial versus fungal challenge (both live and heat-killed). Most, including the canonical AMPs (comprising cecropins, defensins, gambicins, holotricin and attacin), as well as the type F glycine-rich putative AMPs, respond more robustly to bacterial challenge. In *Ae. aegypti*, however, three type D glycine-rich putative AMPs (AAEL021929, AAEL025531 and AAEL025126) are disproportionately responsive to fungal challenge (figure 3*c*). Notably, and in contrast to the insect model *Drosophila*, neither *Ae. aegypti* nor *An. gambiae* mounted specific responses to Gram-negative versus Gram-positive bacteria. Rather, the amplitude of the transcriptional response to bacterial infection appeared roughly proportional, in most cases, to the virulence of the bacterium, as measured by host mortality [64].

### (e) How are the transcriptional responses to bacteria and fungi controlled?

**Figure 4.**
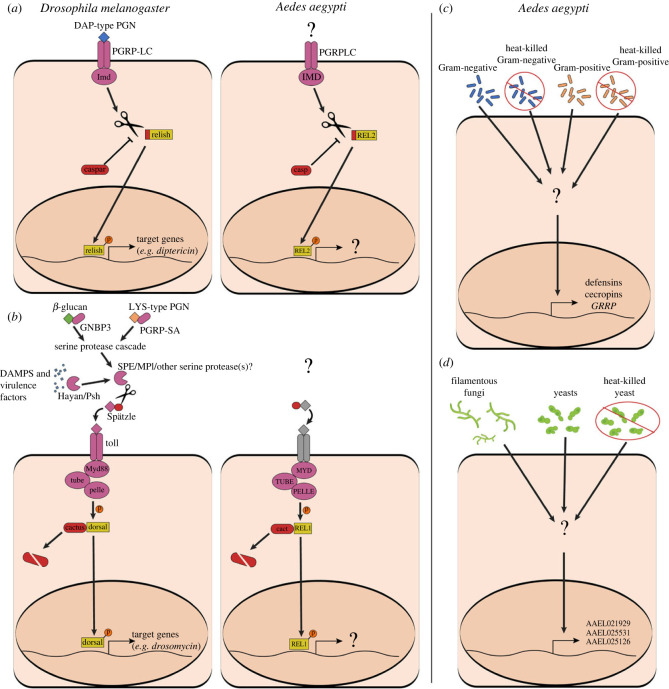
Immune signalling pathways and the specific transcriptional responses of *Aedes aegypti* mosquitoes to bacterial and fungal challenge. (*a*) Simplified representation of the Imd signalling pathway in *Drosophila melanogaster* (left) and *Ae. aegypti* (right). (*b*) Simplified representation of the Toll signalling pathway in *D. melanogaster* (left) and *Ae. aegypti* (right). Receptors and transducers are depicted in purple; inhibitors and inhibitory domains in red; terminal transcription factors in yellow. *Aedes aegypti* possesses many genes encoding spaetzles and Toll receptors (in grey); the exact genes that mediate the initiation of Toll signalling in the context of infection remain uncertain. The precise molecular signals that activate Imd and Toll signalling remain undetermined in mosquitoes. While many genes have been shown to be transcriptionally regulated by *Rel2* and *Rel1* in mosquitoes, there is still uncertainty over which genes are specifically regulated by one pathway versus the other. (*c*) Live and heat-killed bacteria (including Gram-negative and Gram-positive species) promote disproportionate (as compared to fungi) upregulation of defensins, cecropins and holotricin (*GRRP*) in *Ae. aegypti* by an undetermined signalling pathway. (*d*) Fungal pathogens promote disproportionate (as compared to bacteria) upregulation of glycine-rich putative antimicrobial peptides by an undetermined signalling pathway.

Canonically, the differential expression of separate cohorts of AMPs in insects is the product of differential activations of the Imd and Toll immune signalling pathways, mediated by specific recognition of pathogen-associated molecular patterns (figure 4*a,b*). In the *Drosophila* model, the Imd pathway is activated by the binding of the PGRP-LC receptor to DAP-type peptidoglycan [77,78] (characteristic of Gram-negative bacteria) while the Toll pathway is specifically activated by Lys-type peptidoglycan [79] (characteristic of Gram-positive bacteria) and β-glucan [80] (characteristic of fungi). Toll signalling is also more generally activated by danger signals associated with microbial virulence and host damage [81–84]. To our knowledge, however, none of these triggers (DAP-PGN, Lys-PGN, β-glucan, danger signals) have been specifically validated in connection with either pathway in any mosquito species. Further, the lack of distinction in the transcriptional responses of *Ae. aegypti* and *An. gambiae* to Gram-negative versus Gram-positive pathogens [64] casts doubt on the wisdom of extrapolating the canonical model of Imd and Toll activation directly from fruit flies to mosquitoes. Indeed, the validity of the canonical model of immune regulation has been questioned by researchers in mosquito systems. For example, Ramirez *et al*. reported the upregulation of Imd pathway-related genes in *Ae. aegypti* following challenge with filamentous fungal pathogens and posited Imd activation by fungi [85], while Zou *et al*. report that the terminal transcription factors of the Imd and Toll pathways (REL2 and REL1, respectively) target a common cohort of genes, and upregulate them in a synergistic manner [86].

In the absence of validated transcriptional targets exclusively upregulated by one pathway or the other, it is difficult to definitively measure Toll and Imd activation, or to establish their relative involvement in the response to different types of infection (e.g. bacterial versus fungal). While the upregulation of pathway-related genes (e.g. *PGRPLC* or *REL2* for Imd, or *cact* for Toll) is suggestive, transcriptional cross-talk between the two pathways (e.g. the upregulation of Toll pathway genes by Imd signalling, or vice-versa) or regulation of these genes by other mechanisms has not been ruled out. Likewise, with only incomplete pathway knockdowns, it is often difficult to disentangle what portion of the transcriptional response to a given infection is attributable to one pathway or another. However, by comparing transcription patterns across multiple types of infection, it is possible to identify genes that are specifically modulated more by one type of stimulus than another. As mentioned previously, our recent RNAseq study compared the transcriptional responses of *Ae. aegypti* and *An. gambiae* mosquitoes following systemic bacterial and fungal infection [64]. In this analysis, we identified cohorts of genes in both species which are disproportionately upregulated by challenge with live and heat-killed bacteria (mainly defensins and cecropins) and another cohort in *Ae. aegypti* which is disproportionately upregulated by live and heat-killed fungi (type D glycine-rich putative AMPs; figure 4*c,d*). Pairing this observation with reports that, in *Ae. aegypti*: (i) *REL2* overexpression promotes a more robust upregulation of defensins and cecropins when compared with *REL1* overexpression [86], (iii) *REL2* knockdown substantially delays cecropin and defensin expression in infected mosquitoes [87], and (iii) type D glycine-rich putative AMPs are sharply upregulated following silencing of the Toll repressor *cact* [88], we conclude that the bacteria-responsive and fungus-responsive gene cohorts are, most likely, the disproportionate targets of Imd and Toll signalling, respectively. This, in turn, lends supports to a model where the bacterial infection response of mosquitoes is chiefly mediated by the Imd pathway, while Toll signalling is the primary mediator for the response to fungal infection.

## 5. Conclusion

The study of host–microbe interactions in mosquitoes is often dominated by their role as vectors and their interaction with human pathogens. However, bacterial and fungal microbiota play important roles in the biology of mosquitoes, affecting development, digestion, reproduction, pathogen resistance and, crucially, vector competence. Research into these interactions has opened promising avenues for disease transmission control, via paratransgenesis and *Wolbachia* infection, as well as population control via applications of entomopathogenic microbes. Mosquitoes, meanwhile, exert selective pressure on their microbes to shape and corral them in the gut, and to prevent and control systemic infection in the haemocoel. New research has uncovered specific responses to bacterial versus fungal pathogens in the systemic immune response, probably mediated by the Imd and Toll immune-signalling pathways, respectively.

## Data Availability

This article has no additional data.
